# Descriptive Bacterial and Fungal Characterization of Propolis Using Ultra-High-Throughput Marker Gene Sequencing

**DOI:** 10.3390/insects10110402

**Published:** 2019-11-12

**Authors:** Jose F. Garcia-Mazcorro, Jorge R. Kawas, Alicia G. Marroquin-Cardona

**Affiliations:** 1 MNA de Mexico, Research and Development, San Nicolas de los Garza, Nuevo Leon 66477, Mexico; josegarcia_mex@hotmail.com; 2 Faculty of Agronomy, Universidad Autonoma de Nuevo Leon, General Escobedo, Nuevo Leon 66050, Mexico; jorge.kawas@mnademexico.com; 3 Faculty of Veterinary Medicine, Universidad Autonoma de Nuevo Leon, General Escobedo, Nuevo Leon 66050, Mexico

**Keywords:** *Apis mellifera*, bees, honeybees, propolis, microbiota

## Abstract

Bees harbor microorganisms that are important for host health, physiology, and survival. Propolis helps modulate the immune system and health of the colony, but little information is available about its microbial constituents. Total genomic DNA from samples of natural propolis from *Apis mellifera* production hives from four locations in Mexico were used to amplify a region of the 16S rRNA gene (bacteria) and the internal transcriber spacer (fungi), using PCR. The Illumina MiSeq platform was used to sequence PCR amplicons. Extensive variation in microbial composition was observed between the propolis samples. The most abundant bacterial group was *Rhodopila* spp. (median: 14%; range: 0.1%–27%), a group with one of the highest redox potential in the microbial world. Other high abundant groups include *Corynebacterium* spp. (median: 8.4%; 1.6%–19.5%) and *Sphingomonas* spp. (median: 5.9%; 0.03%–14.3%), a group that has been used for numerous biotechnological applications because of its biodegradative capabilities. *Bacillus* and *Prevotella* spp. alone comprised as much as 88% (53% and 35%, respectively) of all bacterial microbiota in one sample. *Candida* (2%–43%), *Acremonium* (0.03%–25.2%), and *Aspergillus* (0.1%–43%) were among the most abundant fungi. The results contribute to a better understanding of the factors associated with the health of *Apis mellifera* production hives.

## 1. Introduction

Honeybees are fascinating eusocial insects of worldwide importance because of their honey production and pollination activities. Honeybees collect diverse materials from surrounding flora, mix it with beeswax (wax produced by the bees, consisting of fatty acids and alcohols) and salivary enzymes, and transport this mixture in their pollen baskets to the hive [1]. This resinous mixture is known as bee glue or propolis and is used by the bees to provide stability to the hive and prevent putrefaction, as well as modulate the immune system and health of the colony [1,2].

Propolis has aroused scientific and medical interest due to its supposed beneficial properties. Hundreds of different chemical compounds have been found in propolis [3], which are thought to be responsible for the observed effects of propolis on health [4,5]. One study showed that the antibacterial, antifungal, and antiviral activity of different propolis were similar in spite of the high differences in their chemical composition [6], suggesting that other unknown factors, aside from chemical composition, could be responsible for the bioactivity of propolis. Other authors also agree with the hypothesis that different propolis chemistry leads to similar biological activity [5].

Like other insects, honeybees carry different microorganisms (microbiota) inside their digestive tracts and in other internal and external tissues [7]. There is an urgent need for research efforts to fully understand the highly dynamic nature between the bees, their microbiota, and environmental factors [8]. This is important because of the multiple beneficial actions of the bees and their susceptibility against environmental pollution [9], and also because microbial life is intimately related to many biological functions in the insect world [10]. While several studies have shown that bees and their hives harbor a rich group of diverse microorganisms [7,11], the microbiological composition of propolis remains poorly understood.

One study published by Grubbs et al. [12] used fatty acid methyl ester and phospholipid-derived fatty acid analysis to profile microbial communities, showing that propolis had the highest microbial community richness (i.e., number of different species) compared to honey, comb, and even the bees themselves [12]. However, this type of analysis is known to be limited to fully describe microbial communities compared to DNA-sequencing efforts, mainly because of the nature of the data obtained (i.e., lipid profiles vs. nucleotides of semi-conserved genes that can be used to infer relationships among microbes and predict metabolic profiles) [13]. In fact, the authors did not determine the composition of the propolis microbiota; instead, they only reached a higher level of classification (e.g., Bacteria, Fungi). Another more recent study used traditional sequencing of PCR amplicons to study bacterial isolates from honey and propolis from *Heterotrigona itama* [14], but this approach is also known to be limited to fully describe complex microbial ecosystems. This current study complements these results by using ultra-high-throughput sequencing of 16S rRNA and internal transcriber spacer (ITS) genes to determine the microbial composition of natural propolis.

## 2. Materials and Methods

### 2.1. Propolis Samples

Because antimicrobials and other drugs are commonly used in beekeeping [15], and different species of bees harbor distinct microbial communities [7], we sought to obtain natural samples of propolis from production hives that did not use antimicrobial treatments and contained the same type of honeybee (i.e., *Apis mellifera*) (see Table 1). Each donated sample represented an aggregate of harvested propolis; therefore, it is likely that the samples represent an aggregate from multiple different zones inside one or more hives. One factor that we could not control was the type of surrounding vegetation, and this is important because it has been suggested that the chemicals (and perhaps microorganisms, too) in propolis are mostly derived from plants [1]. We also did not collect information about the specific subspecies of *A. mellifera*, and this is important because speciation in insects is likely to promote different microbial communities.

### 2.2. Total Genomic DNA Extraction

The donated propolis samples (~20 g each) could not be mixed homogeneously, and therefore we had to rely on subsamples that did not necessarily represent all the original sample. A sample of 5 g of each sample of propolis was frozen at −20 °C, fractioned into small pieces, and placed in a Whatman filter paper No. 1. The filter paper was then placed into a funnel and washed three times, using hexane. All propolis samples were then air-dried in a biosafety level 2 cabinet with laminar flow and EPA filter and stored at −20° C, until further processing, as described elsewhere [16]. For DNA extraction and purification, we used the plant extraction protocol of the Wizard^®^ Genomic Kit (Promega, Madison, Wisconsin, USA), with minor modifications [17]. Briefly, 2 g of wax-free propolis sample in 15 mL Falcon tubes was frozen at −150° C in a conventional freezer for 20 min and grinded up using a sterile mortar. A total of 80 mg of this propolis powder was transferred to a 1.5 mL microcentrifuge tube and mixed with 100 mg of zirconia/silica pellets (0.1 mm diameter) and 600 μL of lysis solution. The mixture was then mixed in a Fast Prep-24^®^ (MP) for 10 s and incubated at 65° C for 15 min. The rest of the DNA-extraction protocol was followed according to the manufacturer’s instructions. DNA concentrations and purity were assessed via a Traycell (Hellma^®^Analytics) in a Shimadzu spectrophotometer. A negative control was included, as usual, to rule out contamination of reagents, and DNA concentrations were adjusted to the minimum concentration obtained.

### 2.3. Analysis of Bacterial Microbiota

Total genomic DNA samples were used to amplify a ~300 bp amplicon, spanning the semiconserved V4 region of the 16S rRNA gene, using primers 515F (5′-GTGCCAGCMGCCGCGGTAA-3′) and 806R (5′-GGACTACHVGGGTWTCTAAT-3′), as described by our group and others [18,19,20]. Sequencing was carried out in a MiSeq platform (Illumina, San Diego, California, USA) at the Molecular Research LP (Shallowater, Texas, USA), following the manufacturer’s instructions. Unless otherwise stated, QIIME [21] v. 1.8.0 was used for all analyses using default scripts. A closed OTU-picking approach was used to assign 16S gene sequences to OTUs, and the generated OTU table was used to calculate alpha diversity indexes. Regarding bacterial taxonomic assignments, the sequencing laboratory (Molecular Research LP) provides a taxonomy assignment of OTUs based on a curated database derived from GreenGenes, RDPII, and NCBI. In our experience, this approach often offers a more precise taxonomy assignment, and therefore these results were used to describe the taxonomic bacterial composition of our propolis samples.

Ultra-high-throughput marker gene sequencing can offer information about the microbial constituents of environmental samples, but the phenotype of the bacteria is difficult to assess. In an effort to predict the functional composition of the bacterial communities in the propolis samples, we used the computational approach Phylogenetic Investigation of Communities by Reconstruction of Unobserved States (PICRUSt) [22].

### 2.4. Analysis of Fungal Microbiota

The same genomic DNA samples from propolis that were used to describe the bacterial microbiota were also used to amplify the internal transcriber spacer (ITS) regions ITS1 and ITS2, using primers ITS5 (forward primer, 5′-GGAAGTAAAAGTCGTAACAAGG-3′, position 1737-1758) and ITS4 (reverse primer, 5′-TCCTCCGCTTATTGATATGC-3′, position 2390-2409) from the Assembling the Fungal Tree of Life (AFToL) project [23,24,25], to investigate the fungal microbiota. As the reference sequences for ITS fragments, we used the UNITE (Unified system for the DNA-based fungal species linked to the classification, [26]) and the Findley database [27]. As with the analysis of the bacterial microbiota, a closed OTU-picking approach was used to assign ITS sequence fragments to OTUs, and the generated OTU table was used to calculate alpha diversity indexes.

### 2.5. Microbial Diversity Analyses

Various indices of richness and diversity were calculated in QIIME, including richness (i.e., the number of phylotypes or OTUs); the nonparametric estimator of diversity Chao1, which is an accurate estimate of richness; the Simpson index, which reflects the probability that any two given organisms sampled will be the same phylotype; and the Shannon–Weaver index, which is a measure of entropy [28]. Sequence data (bacterial and fungal) is freely available at the Sequence Read Archive (NCBI, Bioproject: PRJNA481122).

## 3. Results

### 3.1. Bacterial Microbiota

**Figure 1:**
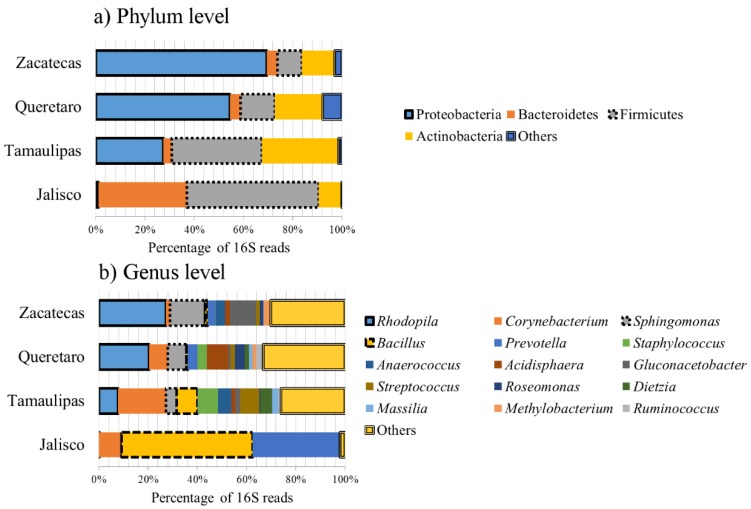
Composition of bacterial microbiota in propolis samples at the phylum (**a**) and genus (**b**) level. Note the high abundance of “other” bacteria (i.e., other bacterial genera not included in this figure because of sake of space) at the genus level. Some groups are highlighted to ease visual representation.

The DNA-extraction procedure produced enough total genomic DNA for its amplification by PCR from all samples (Jalisco: 53.7 ng/μL, Queretaro: 96.2 ng/μL, Tamaulipas: 63.4 ng/μL, and Zacatecas: 121.7 ng/μL; 260/280 ratios for all samples: 1.0–1.1) and negative controls did not generate any absorbance at 260 nm. A total of 100,250 good-quality 16S gene sequences (lowest: propolis from Tamaulipas with 19,367 sequences; highest: propolis from Zacatecas with 29,773 sequences) were analyzed and assigned to 992 OTUs, using the closed OTU-picking approach described above. These numbers of sequences per sample are common in other studies from our research group and others [19,20]. Accordingly to the analysis provided by the Molecular Research LP, the sequences in our dataset were classified into four main bacterial phyla: Proteobacteria (min: 1% Jalisco, max: 69.5% Zacatecas), Bacteroidetes (min: 3.3% Tamaulipas, max: 35.8% Jalisco), Firmicutes (min: 9.9% Zacatecas, max: 53.7% Jalisco), and Actinobacteria (min: 9.3% Jalisco, max: 31.4% Tamaulipas) (see Figure 1).

At lower taxonomic levels, more than 100 different bacterial groups from four main phyla were detected in the propolis samples, showing an unexpected extensive variation in bacterial composition (Figure 1, Table 2). For instance, the 50 most abundant bacterial groups only accounted for ~56% of all sequences (calculated by adding up all median abundances from each genus). At the genus level, the most abundant group was *Rhodopila* spp. (median: 14%; range: 0.1%—27%), a clade that encompass bacteria with one of the highest redox potential (i.e., tendency to acquire electrons) among all microbial life [29].

*Snodgrassella alvi* (family Neisseriaceae, Betaproteobacteria) and *Gilliamella apicola* (family Orbaceae, order Orbales, a sister taxon to the Enterobacteriales, Gammaproteobacteria) are two proposed specialized gut symbionts in the bee’s gut [30] that were originally characterized by using culture techniques [31]. The genus *Snodgrassella* was not found in our dataset, but the family Neisseriaceae was found in three out of four samples, albeit in very low proportions (0.4% in one sample and 0.004% in the other two samples). *Gilliamella* was only found in one sample at a very low abundance (0.02%), being the only representative of the family Orbaceae, a taxon proposed to be related to the Enterobacteriales (Gammaproteobacteria), based on conserved, single-copy amino acid sequences [31].

Other groups of interest include *Sphingomonas* spp., a widely utilized taxon in biotechnology because of its biodegradative properties [32,33,34]. In this study, *Sphingomonas* was present in high abundance in three out of the four propolis samples (Table 2), suggesting that this taxon may be part of the core microbiota present in propolis. Staphylococcus was present in high amounts in two of the four samples (Table 2). Interestingly, only two genera (*Bacillus* and *Prevotella*) comprised ~88% of all bacterial microbiota in Jalisco’s sample, thus highlighting the wide variation in bacterial composition among the samples of propolis.

**Figure 2:**
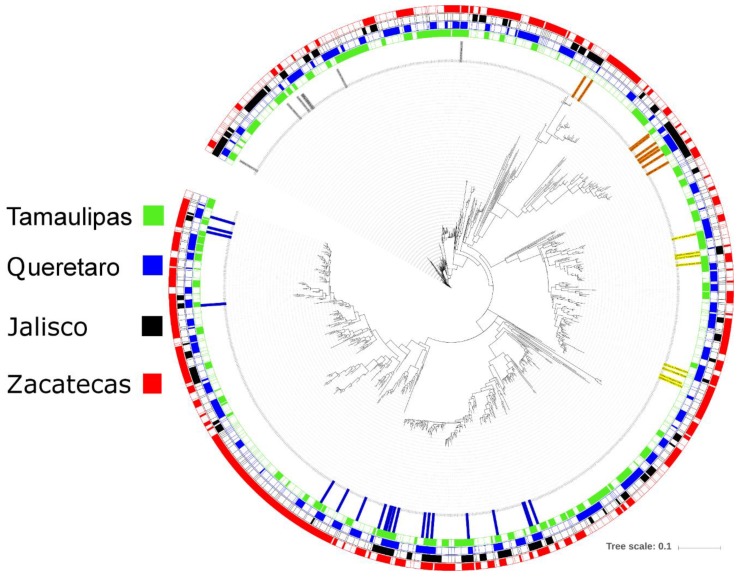
Phylogenetic circular tree of 16S rRNA gene sequences from all 992 OTUs detected in the bacterial microbiota. The tree was built using one representative sequence per OTU, and tree scale refers to sequence dissimilarity (the longer the scale, the bigger the dissimilarity). The four more external (from outside to inside) colored labels denote different locations and show that each propolis sample harbored a distinctive set of OTUs (i.e., only 44 OTUs, or 4% of all OTUs, were present in all propolis samples). The ID of these 44 OTUs that were present in all propolis samples were also colored in the inner row of labels, in accordance with Figure 1: Proteobacteria (20 sequences): blue; Bacteroidetes (9 sequences): orange; Firmicutes (7 sequences): gray, and Actinobacteria (8 sequences): yellow.

In order to obtain a visual representation of the bacterial microbiota, we obtained one representative sequence from each one of the 992 OTUs detected, aligned the sequences in QIIME using PyNAST [35] and built a phylogenetic tree using FastTree [36]. The tree was uploaded into iTOL [37] for visualization and annotation (see Figure 2). This analysis revealed the each propolis sample seemed to harbor different bacterial taxa, with only 44 of all 992 OTUs (or ~4%) that were present in all samples (7 from Firmicutes, 9 from Bacteroidetes, 8 from Actinobacteria, and 20 from Proteobacteria) (Figure 2). Together with the taxonomic information that we obtained, these results also point out to a role of Proteobacteria in propolis and likely inside the hives that deserve more investigation.

### 3.2. Predicted Metabolic Profile of Bacterial Microbiota

PICRUSt analysis revealed that the lower diversity of bacteria in the propolis sample from Jalisco may have significantly impacted the predicted metabolic profile (i.e., the sample from Jalisco showed the highest difference compared to the overall median for most features) (Table 3). Interesting exceptions included genes related to the phosphotransferase system (higher in Tamaulipas), transcription factors (lower in Zacatecas), and arginine and proline metabolism (lower in Tamaulipas).

### 3.3. Fungal Microbiota

A total of 113,644 good-quality sequences (lowest: propolis from Jalisco with 3737 sequences; highest: propolis from Zacatecas with 47,044 sequences) were analyzed and classified into only two phyla, Basidiomycota (min: 6.9%, max: 28.3%) and Ascomycota (min: 71.7%, max: 92.7%). A closed out-picking approach against the UNITE database (reference sequences at both 97% and 99% similarity were used) only yielded 2—3 OTUs. In contrast, the results using the Findley reference sequence database (23,456 sequences) revealed the presence of 39 (forward reads) and 34 (reverse reads) OTUs from different fungal organisms (Table 4). *Candida*, *Acremonium*, and *Aspergillus* were the most abundant fungal organisms in our samples, and, similarly to the bacterial microbiota, Jalisco’s sample displayed the lowest diversity of fungi, with only two genera (*Candida* and *Starmerella*) comprising the vast majority of sequences (43% each, together comprising ~86%, Table 4).

**Figure 3:**
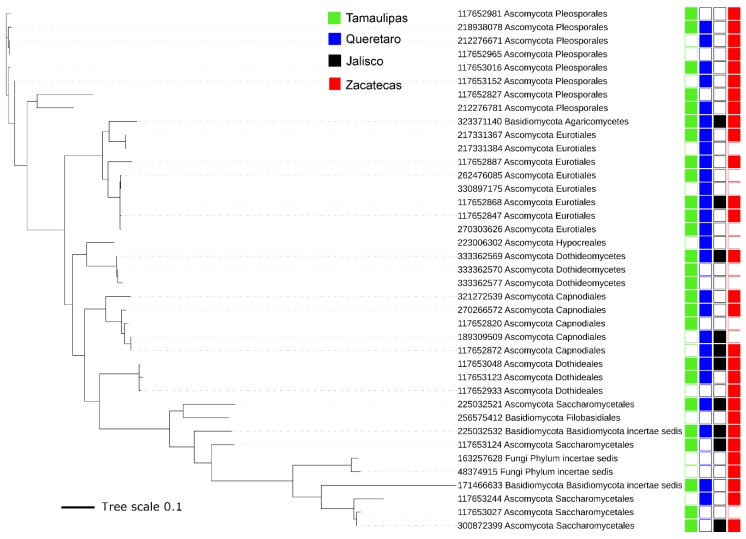
Phylogenetic tree of ITS gene sequences from all 39 OTUs detected in the fungal microbiota. The tree was built using one representative sequence per OTU, and tree scale refers to sequence dissimilarity (the longer the scale, the bigger the dissimilarity). The numbers written in the same taxa names refer to the IDs in the Findley database.

We attempted to perform a similar approach of visualizing the clustering of sequences from the fungal microbiota to the one we took with the bacterial microbiota. Therefore, we obtained a set of representative sequences from each one of the 39 OTUs, but we could not align these sequences using PyNAST or any other alignment method available in QIIME. To overcome this, we tried to first align the Findley reference sequences in order to supply this alignment as a template alignment in QIIME; however, we also could not align the 23,456 sequences in this file. Successful alignment was accomplished using ClustalW in DAMBE [38] with default settings. The FastTree method in QIIME was used to build the phylogenetic tree for analysis, using iTOL (Figure 3). Similar to the results obtained from the bacterial microbiota, only a few OTUs (6 of 39, or ~15%) from fungal organisms were detected in all propolis samples (Figure 3). Note that this percentage is higher compared to the percentage of bacterial OTUs that were present in all samples (~4%), perhaps reflecting a difference in ubiquitousness between bacteria and fungi in propolis.

### 3.4. Microbial Diversity

Diversity analyses revealed intriguing results (Table 5). For instance, while the global fungome is estimated to comprise at least six million different species [39], in this study, the number of OTUs in the fungal microbiota was about 10 times less compared to the OTUs in bacterial microbiota (Table 5). To investigate whether another, more flexible, OTU-picking approach would reveal useful information about the diversity of fungal organisms in the propolis, we used an open approach that allows unassigned reads to cluster into new OTUs. This approach theoretically can provide a wider view of the true diversity of sequences, and, in our experience, it works well on 16S rRNA gene sequences. Using the Findley sequence database, we discovered a total of 156 (forward reads) and 1418 OTUs (reverse reads), but these numbers are still lower compared to the bacterial OTUs normally found in the animal gut when using the same open OTU-picking approach [19,20].

## 4. Discussion

Honeybees are fascinating insects of worldwide importance. Propolis is used by the bees to provide stability to the hive, prevent putrefaction, and modulate the health of the colony. Two studies have investigated the microbiota in propolis by using fatty acid analysis, culture techniques, and traditional sequencing [12,14], but these approaches are known to be limited for providing a comprehensive view of complex microbial ecosystems. This paper complements these two studies and shows for the first time the bacterial and fungal inhabitants of natural propolis, using high-throughput sequencing.

Honey and propolis have historically been considered to be relatively aseptic [12], which, in honey, has been explained by its high osmolarity, acidity, and the presence of antibiotics [40]. However, a complex, metabolically active microbiome has been discovered in honey [41]. While it is not our objective to discuss the asepsis of propolis or honey, it is important to recall that sequencing metagenomic efforts (such as the one presented in this current communication) only detect genetic sequences which can be derived from dead and live microorganisms [42], and the identification of either one is challenging to determine with accuracy. In the case of dead microorganisms, it is even more difficult to determine at what point in the past they were metabolically active. This is an area that deserves scrutiny in bee science because at least three sources of the chemical compounds in propolis have been suggested: plant materials, substances from bee metabolism, and materials that are introduced accidentally during propolis elaboration and collection by the bees [1]. It is likely that these three are also sources of the microorganisms present in propolis. Note that great progress has been achieved in determining the routes of acquisition of the bees’ gut microbiota [43].

Ngalimat et al. [14] offered interesting insights into the live microbiota in propolis, honey, and bee bread. Using culture techniques, they isolated a total of 41 different bacterial types and showed that their 16S genes were related to Firmicutes (37 isolates), Proteobacteria (three isolates) and Actinobacteria (one isolate). In this current study, these phyla were also the most predominant ones, with a minor contribution of Bacteroidetes in most samples (Jalisco: 35.8%, rest of the samples: 3%—4%). Even though the numbers of Bacteria were very low (< 6 × 10^4^ colony-forming units/g of product) in that study [14], it may still be possible to obtain more isolates, perhaps using other culture media and conditions, to evidence the presence of low abundant microbes. Overall, the congruence between our results and the results offered by Ngalimat et al. strongly suggest that sequencing efforts offer a good proxy for the microbiota living in propolis and other bee products. However, the exact proportion of genomic sequences that come from dead microorganisms still requires a more precise determination.

Propolis is a challenging environment to thrive because of its physical and chemical characteristics. Therefore, we speculate that the survival and proliferation of microbes in propolis may have to do with specialized respiratory chains that transfer electrons from electron donors to electron acceptors via redox reactions, a complex biological phenomenon that relies on organic compounds such as quinones. In this regard, the genus *Rhodopila* (a group with one of the highest redox potentials in the microbial world) was found to be highly abundant in three of the four samples, but the main quinones in this taxon have not been investigated. To our knowledge, *Rhodopila* has not been detected in animal gut, in bees, in beehives, or in plant materials or exudates, but it has been found in epilithic biofilms [44] and sphagnum mosses [45]. Another study showed the presence of 16S sequences that were similar to *Rhodopila globiformis* in a moorland soil that is highly polluted with polychlorinated biphenyls [46]. More research is necessary to determine the biological characteristics of *Rhodopila* present in propolis.

*Snodgrassella alvi* and *Gilliamella apicola* are two proposed specialized gut symbionts in the bee’s gut [30]. The fact that both bacteria were not found to be highly abundant in our propolis samples may imply that the bacteriological composition of propolis differs from the bee-associated microbiota. Interestingly, both of these bacteria were shown to have ubiquinone-8 as their major respiratory quinone [31]. Unlike *Snodgrassella* and *Gilliamella*, *Sphingomonas* (which in this study showed a high abundance in 3 of 4 samples) has ubiquinone-10 as its major type of ubiquinone [47]. This is important because different Bacteria may rely on different types of quinones to thrive.

The sample from the state of Jalisco was the most intriguing one to us, due to the predominance of only two major bacterial taxa, *Bacillus* (53%) and *Prevotella* (35%), which together accounted for almost 90% of all taxa. The location in Jalisco was the only one that had *Agave tequilana* in the surrounding flora, but whether this is related to the taxonomy and much lower diversity of propolis, bacterial microbiota needs further studies. Also, it was not possible to know whether the bees preferred this or the other plants (e.g., *P. granulosa*). Other factors that may be related to this peculiar bacterial composition include the chemical composition and corresponding antimicrobial activity, which is highly dependent on geographical origin and plant source [48]. Moreover, it is also possible that different subspecies of *A. mellifera* carry different microbial communities. Aside the different flora, there was no other indication that could make this sample different from the others, and therefore the high abundance and potential cooperation between *Bacillus* and *Prevotella* in propolis remains to be studied.

Fungi inhabit many environments in the world, but the study of fungal microbiomes is hampered by several important challenges [49]. For instance, ITS regions are highly variable in size and composition and have been shaped by different evolutionary forces compared to ribosomal gene markers. Even though the ITS regions are considered to be the universal barcode for fungi [25], there still remains doubts about its usefulness to fully describe fungal communities. The study of the fungal microbiota of propolis is important because some fungi (i.e., *Candida* and other yeasts such as *Saccharomyces*) have been suggested to play an important role in the digestion of substrates and in detoxification of toxic plant metabolites in the insect host [50], and some varieties of honey have been shown to possess an antifungal effect against *Candida* species [51,52]. On the other hand, *Starmerella* has received increased attention in the wine industry [53] and has been discovered in stingless bees [54,55].

Another interesting finding of this descriptive study was the lower diversity of fungal organisms compared to bacteria. Meason-Smith et al. [56] also revealed a very low diversity of fungi (albeit a little higher compared to our numbers) in diverse anatomical sites in dogs (~5—110 OTUs), using a similar approach. This is important to highlight because the gut provides an environment that likely contributes to the proliferation of more types of organisms. Overall, it is likely that either ribosomal, ITS, or any other potential gene marker cannot fully determine the microbial communities in a given environment. Overall, we think that either our results underestimate the true diversity of fungi in propolis samples, or that propolis truly possesses a much lower diversity of fungi compared to bacteria. In an attempt to answer these questions, we used an open OTU-picking approach that theoretically can provide a wider view of the true diversity of sequences. However, the numbers of OTUs obtained from this approach were still much lower compared to the bacterial OTUs normally found in the animal gut when using the same open approach. More research, perhaps using other gene markers, may help discover the true diversity of fungal organisms in propolis and other environments.

## 5. Conclusions

In summary, there is an urgent need for multidisciplinary research efforts to fully understand the highly dynamic nature between the bees, their microbiota, and environmental factors inside and outside the hives. This study complements other studies that have looked at the microbial composition in samples of propolis. The most abundant bacterial group, *Rhodopila*, has not been found in any bee-related environment, thus suggesting that propolis may serve to favor the growth of certain types of microorganisms that are not necessarily highly abundant in surrounding environments (e.g., plant materials, the bee host, the hive). The survival and proliferation of microorganisms in propolis is likely related to specialized respiratory chains. Clearly, more studies on the contribution of the propolis microbiota to the health of bees are warranted.

## Figures and Tables

**Table 1:** Characteristics of the locations from which we obtained the propolis samples.

State	Location	Principal Flora
Jalisco	Santa Anita, Tlaquepaque LAT/LONG 20.55/103.45	Blue maguey (*Agave tequilana*) and mesquite (*Prosopis granulosa*)
Queretaro	Conca, Arroyo Seco LAT/LONG 21.43/99.67	Coffee (*Coffea arabica*) and mesquite (*Prosopis granulosa*)
Tamaulipas	El Carmen, Guemez LAT/LONG 24.07/99.13	Orange trees (*Citrus sinensis*) and mesquite (*Prosopis granulosa*)
Zacatecas	Fresnillo LAT/LONG 32.17/102.88	Mesquite (*Prosopis granulosa*)

**Table 2:** Relative abundances of the 25 most abundant bacterial taxa (genus level) found in the propolis samples^1^.

Bacterial Group	Jalisco	Queretaro	Tamaulipas	Zacatecas	Overall Median
*Rhodopila*	0.08%	20.3%	7.8%	27.3%	14.1%
*Corynebacterium*	9.1%	7.7%	19.5%	1.6%	8.4%
*Sphingomonas*	0.03%	7.7%	4.3%	14.3%	5.9%
*Bacillus*	53.3%	0.5%	8.5%	1.0%	4.8%
*Prevotella*	35.3%	4.1%	0.1%	3.1%	3.5%
*Staphylococcus*	0.06%	3.9%	8.2%	0.1%	1.9%
*Anaerococcus*	0.03%	0.01%	5.5%	3.9%	1.9%
*Acidisphaera*	0.01%	8.8%	1.5%	1.9%	1.6%
*Streptococcus*	0.02%	1.6%	7.8%	1.5%	1.5%
*Gluconacetobacter*	0.02%	1.0%	2.1%	10.8%	1.5%
*Roseomonas*	0.01%	4.1%	0.3%	1.5%	0.9%
*Dietzia*	0.01%	1.8%	4.8%	0.00%	0.9%
*Massilia*	-	1.4%	2.6%	0.2%	0.8%
*Methylobacterium*	0.00%	2.9%	1.1%	0.4%	0.8%
*Ruminococcus*	0.00%	2.9%	1.1%	0.4%	0.8%
*Ornithinibacillus*	0.01%	1.3%	1.7%	0.00%	0.67%
*Microbacterium*	0.00%	1.3%	-	1.3%	0.62%
*Curvibacter*	0.50%	0.80%	0.53%	0.57%	0.55%
*Clostridium*	0.01%	0.82%	1.7%	0.05%	0.44%
*Flavobacterium*	0.50%	0.31%	1.9%	0.31%	0.41%
*Curtobacterium*	0.03%	5.3%	0.01%	0.71%	0.37%
*Pseudomonas*	0.02%	3.5%	0.58%	0.08%	0.33%
*Cronobacter*	0.01%	0.60%	3.2%	0.05%	0.32%
*Aquabacterium*	0.01%	0.30%	0.34%	0.43%	0.32%
*Terriglobus*	0.02%	5.5%	0.01%	0.47%	0.24%
All other taxa	~1%	~13%	~16%	~25%	NA

^1^ A dash symbol (-) was used for taxa that was absent (0% abundance), in order to differentiate it from those taxa that showed very low abundance (e.g., *Methylobacterium* and *Ruminococcus* in Jalisco’s sample).

**Table 3:** First 20 most abundant features obtained from PICRUSt analysis from the bacterial microbiota^1^.

Feature in PICRUSt	Jalisco	Queretaro	Tamaulipas	Zacatecas	Overall Median
Membrane transporters	**5.5%**	7.0%	**7.3%**	7.1%	7.0%
ABC transporters	**3.0%**	4.2%	4.3%	**4.3%**	4.3%
Ribosome	**2.7%**	2.1%	2.3%	**2.0%**	2.2%
Peptidases	**2.3%**	1.8%	1.9%	**1.7%**	1.8%
Pyrimidine metabolism	**2.1%**	1.6%	1.8%	**1.5%**	1.7%
Transcription machinery	**1.3%**	0.9%	0.9%	**0.8%**	0.9%
Starch and sucrose metabolism	**1.1%**	0.8%	0.8%	**0.7%**	0.8%
Butanoate metabolism	**0.9%**	1.2%	1.2%	**1.4%**	1.2%
DNA repair and recombination proteins	**3.3%**	2.9%	3.0%	**2.8%**	3.0%
Secretion system	**1.4%**	1.7%	**1.8%**	1.7%	1.7%
Chromosome	**1.7%**	1.4%	1.5%	**1.4%**	1.5%
DNA replication proteins	**1.3%**	1.0%	1.1%	**1.0%**	1.1%
Amino-acid-related enzymes	**1.7%**	1.4%	1.5%	**1.4%**	1.5%
Phosphotransferase system	0.2%	0.3%	**0.5%**	**0.2%**	0.2%
Cysteine and methionine metabolism	**1.2%**	**1.0%**	1.0%	1.0%	1.0%
Peptidoglycan biosynthesis	**1.0%**	0.7%	0.8%	**0.7%**	0.7%
Glutathione metabolism	**0.3%**	0.6%	0.5%	**0.6%**	0.5%
Bacterial secretion system	**0.6%**	0.9%	0.9%	**0.9%**	0.9%
Valine, leucine and isoleucine degradation	**0.8%**	1.0%	0.9%	**1.2%**	1.0%
Two-component system (signal transduction)	**1.9%**	**2.2%**	2.1%	2.1%	2.1%

^1^ The lowest and the highest percentages of each feature are highlighted in bold to ease visualization.

**Table 4:** Relative abundances of all fungal taxa (genus level) found in the propolis samples.

Fungal Group	Jalisco	Queretaro	Tamaulipas	Zacatecas	Overall Median
*Candida*	42.6%	9.5%	22.8%	2.1%	16.2%
*Acremonium*	0.03%	25.2%	10.6%	2.6%	6.6%
*Aspergillus*	0.08%	6.6%	42.6%	2.2%	4.4%
*Phanerochaete*	0.11%	22.3%	3.6%	2.9%	3.2%
*Cladosporium*	1.3%	4.9%	0.65%	2.8%	2.1%
*Starmerella*	42.5%	1.9%	2.1%	0.3%	2.0%
*Monascus*	0.11%	7.2%	2.8%	0.41%	1.6%
*Wallemia*	0.11%	1.1%	1.7%	1.2%	1.2%
*Alternaria*	-	0.39%	2.8%	1.0%	0.71%
*Malassezia*	-	0.01%	2.1%	1.13%	0.57%
*Trichosporonoides*	-	4.2%	0.30%	0.53%	0.42%
*Moniliella*	12.9%	0.76%	0.06%	0.04%	0.41%
*Phoma*	-	0.03%	0.78%	17.6%	0.40%
*Penicillium*	-	0.37%	3.2%	0.06%	0.21%
*Cephalosporium*	0.06%	11.6%	0.05%	0.34%	0.20%
*Ampelomyces*	-	-	0.34%	0.47%	0.17%
*Coniochaeta*	0.08%	0.03%	0.04%	43.8%	0.06%
*Spencermartinsia*	0.06%	0.01%	3.2%	0.07%	0.06%
*Ramulispora*	-	1.8%	0.01%	0.11%	0.06%
*Priceomyces*	-	0.18%	-	0.05%	0.02%
*Hyphopichia*	0.03%	-	0.17%	0.02%	0.02%
*Debaryomyces*	0.03%	-	0.12%	0.02%	0.02%
*Batcheloromyces*	0.00%	0.95%	0.00%	0.04%	0.02%
*Aureobasidium*	0.03%	0.01%	0.01%	10.2%	0.02%
*Eremascus*	-	0.85%	0.01%	0.02%	0.01%
*Exophiala*	0.03%	-	-	0.92%	0.01%
*Dactylella*	0.03%	-	-	2.5%	0.01%
*Knufia*	0.03%	-	-	1.6%	0.01%
*Sclerostagonospora*	-	-	0.01%	0.80%	0.01%
*Cyphellophora*	-	0.00%	-	0.68%	0.00%
*Westerdykella*	-	-	-	0.45%	0.00%
*Kwoniella*	-	-	-	1.06%	0.00%
*Fusarium*	-	0.14%	-	-	0.00%
*Curvularia*	-	-	-	0.10%	0.00%
*Mucor*	-	-	-	0.37%	0.00%
*Cryptococcus*	-	-	-	0.06%	0.00%
*Zeloasperisporium*	-	-	-	1.6%	0.00%

^1^ A dash symbol (-) was used for taxa that was absent (0% abundance) to differentiate it from those taxa that showed very low abundance (e.g., *Batcheloromyces* in Jalisco’s sample).

**Table 5:** Alpha richness and diversity indices for bacterial and fungal microbiota^1^.

Sample	OTUs	Chao1	Shannon	Simpson
Jalisco (fungal)	13	20	1.1	0.51
Tamaulipas (fungal)	28	56	2.2	0.65
Queretaro (fungal)	33	35	2.8	0.81
Zacatecas (fungal)	37	39	2.6	0.69
Jalisco (bacterial)	240	510	1.9	0.61
Tamaulipas (bacterial)	357	663	5.6	0.95
Queretaro (bacterial)	363	611	5.1	0.90
Zacatecas (bacterial)	542	1048	6.0	0.92

^1^ The same closed OTU-picking approach was used for both bacterial and fungal microbiota. Note that the samples were organized from the lowest to the highest numbers of OTUs.
